# Ohmic Heating as a Strategy for Upcycling PET Plastic Waste for New Supramolecular Structures

**DOI:** 10.3390/molecules31203698

**Published:** 2026-10-15

**Authors:** Susana Guimarães, Susana L. H. Rebelo, Sandra Gago, Maria A. Salgado, Luís M. N. B. F. Santos, Salete S. Balula, Luís Cunha-Silva, Ana M. G. Silva

**Affiliations:** 1 LAQV-REQUIMTE, Department of Chemistry and Biochemistry, Faculty of Sciences, University of Porto, 4169-007 Porto, Portugal; up202005190@edu.fc.up.pt (S.G.); susana.rebelo@fc.up.pt (S.L.H.R.); m.balula@fct.unl.pt (S.S.B.); luis.csilva@lneg.pt (L.C.-S.); 2 LAQV-REQUIMTE, Chemistry Department, NOVA School of Science and Technology, NOVA FCT, Campus de Caparica, 2829-516 Caparica, Portugal; s.gago@fct.unl.pt; 3 CIMAR, Instituto de Ciências Biomédicas de Abel Salazar, University of Porto, Rua de Jorge Viterbo Ferreira, 228, 4050-313 Porto, Portugal; msalgado@icbas.up.pt; 4 CIQUP, Institute of Molecular Sciences (IMS), Departamento de Química e Bioquímica, Faculdade de Ciências, University of Porto, Rua do Campo Alegre, 4169-007 Porto, Portugal; lbsantos@fc.up.pt; 5 LNEG— National Laboratory of Energy and Geology IP, Campus do Lumiar, 1649-038 Lisboa, Portugal; 6 LAQV-REQUIMTE, School of Medicine and Biomedical Sciences, University of Porto, Rua de Jorge Viterbo Ferreira, 228, 4050-313 Porto, Portugal

**Keywords:** PET waste, alkaline hydrolysis, terephthalic acid, ohmic heating, ionic/hydrogen-bonding, co-crystals

## Abstract

The massive accumulation of poly(ethylene terephthalate) (PET) plastic waste poses a significant global environmental challenge, requiring the development of more sustainable upcycling strategies. This study investigated the alkaline hydrolysis of PET flakes to recover terephthalic acid (TPA) using ohmic heating and compared its performance with conventional and microwave heating methods. The sequential PET loading in the same reaction medium, as well as the use of opaque white and opaque colored PET feedstocks, was also evaluated. The spectroscopic characterization by ^1^H NMR and powder X-ray diffraction (PXRD) confirmed the successful recovery of TPA from different PET sources. This recovered TPA was subsequently co-crystallized with 4-(dimethylamino)pyridine (DMAP) and 4-aminopyridine (AP) in low-temperature methanol to synthesize new supramolecular structures **1** and **2**, respectively. Both compounds were characterized by scanning electron microscopy (SEM) and PXRD. Single-crystal X-ray diffraction analysis of compound **1** revealed a three-dimensional supramolecular network stabilized by ionic/hydrogen bonding interactions between the TPA and DMAP units. Overall, ohmic heating emerged as a potentially energy-efficient and sustainable approach for PET hydrolysis. Furthermore, the successful synthesis of these supramolecular architectures demonstrates that recycled TPA can serve as a valuable building block for advanced functional materials, highlighting a promising route for the high-value upcycling of PET waste.

## 1. Introduction

Contemporary patterns of consumption generate unprecedented amounts of plastic waste. In the European Union (EU), the annual production of plastic waste is estimated at 16.16 million tons, of which only 40.7% (approximately 6.6 million tons) is successfully recycled [1]. The remaining fraction is typically disposed of in landfills, incinerated or released into natural environments such as oceans, rivers and forests. This situation is becoming increasingly critical as cities and countries develop rapidly without adequate systems to manage the changing composition of citizen waste [2,3].

In Portugal, for instance, only about one-third of plastic packaging is currently recycled, despite the estimated economic value of recycled plastics reaching 167 €/t [4]. These figures highlight both the environmental and economic incentives for expanding recycling capacity. Consequently, considerable efforts have been directed toward developing technologies capable of transforming plastic waste into high-value products. Among these approaches, chemical recycling has attracted increasing attention because it enables the depolymerization of plastics into their constituent monomers, which can subsequently be reused as feedstocks for the production of new materials, thereby reducing dependence on fossil resources [5]. The development of innovative recycling technologies is therefore essential not only for creating value-added materials but also for advancing sustainable development in line with the United Nations Sustainable Development Goals (2030 Agenda) [6,7].

As the primary polymer used in single-use beverage bottles, poly(ethylene terephthalate) (PET) constitutes a significant fraction of global plastic waste. Its accumulation in terrestrial and aquatic ecosystems represents a severe environmental concern, as PET may persist for decades before undergoing significant degradation [8]. While mechanical recycling is highly effective for transparent PET bottles, it faces severe technical limitations when applied to colored or opaque PET bottles, trays, and multi-layered films [9,10]. The presence of dyes, pigments, labels, adhesives, and opacifying agents such as titanium dioxide (TiO_2_) can compromise the quality of recycled products and reduce the efficiency of conventional recycling processes [11]. Consequently, alternative chemical recycling strategies are required for these more challenging PET waste streams.

Chemical recycling enables the depolymerization of PET into its monomers, which can be used as valuable building blocks for preparing new materials, thereby contributing to a more circular use of resources. The main chemical recycling processes for PET include glycolysis, methanolysis, aminolysis and hydrolysis [11]. Glycolysis and methanolysis produce oligomers or monomers, such as bis(hydroxyethyl)terephthalate (BHET) and dimethyl terephthalate (DMT), respectively, whereas aminolysis affords terephthalamide derivatives [2,5]. In contrast, hydrolysis enables the recovery of terephthalic acid (TPA), one of the original monomers used in PET production [2,5]. Among the hydrolysis strategies, alkaline hydrolysis uses aqueous solutions of bases, such as NaOH or KOH, to convert PET into ethylene glycol (EG) and the corresponding terephthalate salt, which is subsequently acidified to recover TPA. An important advantage of this process is its applicability to complex and contaminated post-consumer PET waste streams, enabling the recovery of high-purity TPA through acidification, precipitation, filtration, and washing [12,13]. In the present study, alkaline hydrolysis was selected because of its operational simplicity, its suitability for post-consumer PET feedstocks, and the direct recovery of TPA, which can be reused in new applications [14,15].

Conventional hydrolysis processes rely on conductive heat transfer through reactor walls, which is often associated with long reaction times and limited energy efficiency. Microwave-assisted heating overcomes some of these limitations by generating heat directly within the reaction medium. However, microwave irradiation may result in non-uniform temperature gradients and localized hot spots, making it difficult to scale up because of the limited penetration depth of microwave radiation in reaction mixtures [16]. Electrocatalytic recycling of PET also presents itself as an innovative method for recycling this material, with challenges related to catalyst performance, PET sources, and reactor design [17].

In this context, ohmic heating emerges as a promising alternative [18]. Ohmic heating is an electrothermal process in which an alternating electric current (AC) (where the AC frequency is used in the optimization process) passes directly through the reaction medium, generating heat internally due to the movement of charged species in solution. Since heat is generated directly in the reaction medium, ohmic heating minimizes heat-transfer losses associated with conventional external heating. This enables rapid and energy-efficient heating and offers potential advantages for scale-up toward pilot- and industrial-scale applications [19]. In the present system, two stainless steel electrodes, compatible with alkaline reaction conditions, were used in combination with magnetic stirring, reflux and precise temperature control to ensure effective heating and good homogenization of the reaction mixture.

While this technology is well-established in the food industry [20,21] and has recently been adopted for organic synthesis [22,23], its application to plastic upcycling remains unexplored. Beyond monomer recovery, the chemical recycling of PET can contribute to a circular economy by generating building blocks for advanced functional materials [15,24,25]. In particular, TPA is an attractive precursor for supramolecular chemistry because its carboxylic acid groups can participate in a wide range of non-covalent interactions [26,27,28].

Accordingly, the integration of recovered TPA into supramolecular self-assembly processes offers a potential strategy for converting plastic waste into high-value functional materials. Molecular self-assembly allows the preparation of binary materials with advantages such as mild and noncovalent processing, reproducibility, introduction of nanoscale to mesoscale organization, conductivity, proton transport, and tunable porosity, as well as emerging optical and electronic properties arising from the delocalization of electronic states across many building blocks [29]. These characteristics have enabled the development of functional materials for applications including electrocatalysis, light harvesting, sensing, energy storage, and drug delivery [30,31].

The main objective of this work is to explore the ohmic heating alkaline hydrolysis of PET [32] with the subsequent valorization of the recovered terephthalic acid into supramolecular architectures. The performance of ohmic heating was compared with conventional and microwave heating methods for the alkaline hydrolysis of PET flakes. The robustness of the process was further assessed using transparent, opaque white, and opaque colored PET feedstocks, as well as through reaction-medium reuse experiments involving the sequential addition of fresh PET.

To the best of our knowledge, this is the first report describing the application of ohmic heating to PET depolymerization. In ohmic heating, heat is generated directly in the reaction medium by passing a high-frequency AC electric current, making the electrical conductivity of the solution a key parameter controlling the heating profile. In this process, NaOH plays a dual role, simultaneously providing the ionic conductivity required for ohmic heating and acting as the reagent responsible for PET hydrolysis. Therefore, optimization of its concentration is essential for achieving suitable heating conditions while promoting efficient PET depolymerization.

Finally, the recovered TPA was employed as a supramolecular building block in combination with 4-(dimethylamino)pyridine (DMAP) and 4-aminopyridine (AP), leading to the synthesis and characterization of new three-dimensional architectures stabilized by non-covalent interactions. This approach demonstrates a sustainable pathway for transforming PET waste into value-added functional materials with potential applications in sensing and other advanced technologies.

## 2. Results and Discussion

### 2.1. PET Hydrolysis

**Scheme 1:**
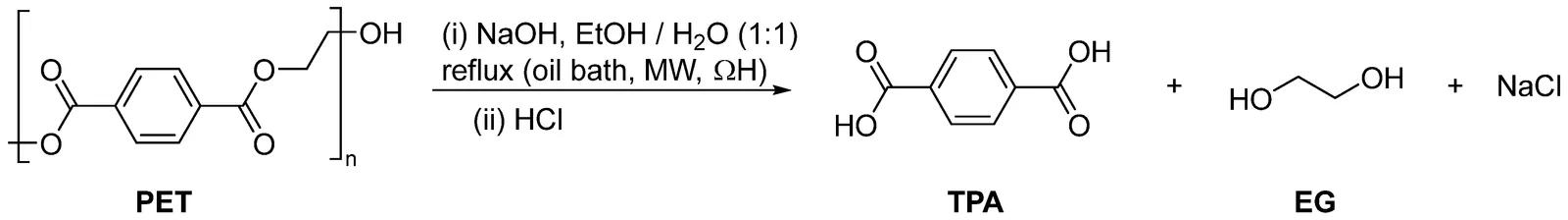
Alkaline depolymerization of poly(ethylene terephthalate) (PET) in the presence of NaOH, EtOH and water, using conventional, microwave and ohmic heating (25 kHz), followed by treatment with HCl, producing terephthalic acid (TPA), ethylene glycol (EG) and NaCl.

Our study began by exploring the alkaline hydrolysis (Scheme 1) of post-consumer transparent PET flakes, using a protocol adapted from Ügdüler et al. [11], where NaOH concentrations of 5, 10, and 15 wt% were evaluated. To do this, clean post-consumer PET bottles were cut and ground to produce PET flakes, which were characterized by particle size distribution analysis (Table S1, see Supplementary Materials). Subsequently, these PET flakes were mixed with 6 wt% NaOH in 50 mL of an EtOH:H_2_O (1:1, *v*/*v*) mixture. Hydrolysis was conducted at reflux (83 °C) using three heating methodologies: conventional, microwave and ohmic heating. For each experiment, PET conversion and terephthalic acid (TPA) yield were calculated according to Equations (1) and (2), respectively [11,33]:

(1) $$PET\;conversion\left(\%\right)=\frac{W_{iPET}-W_{uPET}}{W_{iPET}}\times 100$$

(2) $$TPA\;yield\left(\%\right)=\frac{W_{TPA}}{W_{iPET}\times 0.874}\times 100$$

where W_iPET_, W_uPET_ and W_TPA_ are the weights of raw initial PET, the unreacted PET, and the produced TPA, respectively. The 0.874 coefficient corresponds to the ratio of molar mass of TPA and molar mass of the monomeric unit of PET [33].

The results are presented in Table 1 and reveal that, while conventional and microwave heating methods successfully yielded TPA after 90 min (entries 1 and 2), the initial ohmic heating experiments were unsuccessful (entry 3). Although the efficiency of ohmic heating is strongly dependent on the electrical conductivity of the medium, it was observed that a 6 wt% NaOH concentration results in an excessively conductive medium (72.3 mS cm^−1^). Under these conditions, the system exhibited a significant voltage drop, high current intensity, and severe operational constraints. To overcome this limitation, the NaOH concentration was reduced to 3 wt%. Under these optimized conditions, the ohmic heating process proceeded smoothly, resulting in TPA yields of 51% after 120 min and 85% after 180 min (entries 4 and 5). Increasing the NaOH concentration to 4 wt% allowed for a reduced reaction time of 120 min, providing a TPA yield of 80% (entry 6). Further extending the reaction time to 180 min enhanced the process performance (entry 7). To evaluate the possibility of reducing reagent consumption and waste generation, the reaction medium recovered after PET hydrolysis was reused for the processing of an additional PET load. Under these conditions, an overall TPA yield of 76% was obtained (entry 8), demonstrating the potential of reaction-medium reuse as a sustainable approach for PET depolymerization.

The optimized ohmic heating protocol was subsequently applied to opaque white and opaque colored PET flakes. In both cases, PET depolymerization proceeded successfully, although slightly lower conversion values and TPA yields were observed when compared with transparent PET (entries 9 and 10). These differences are likely associated with the presence of additives commonly used in colored and opaque PET formulations, which may reduce hydrolysis efficiency.

**Figure 1:**
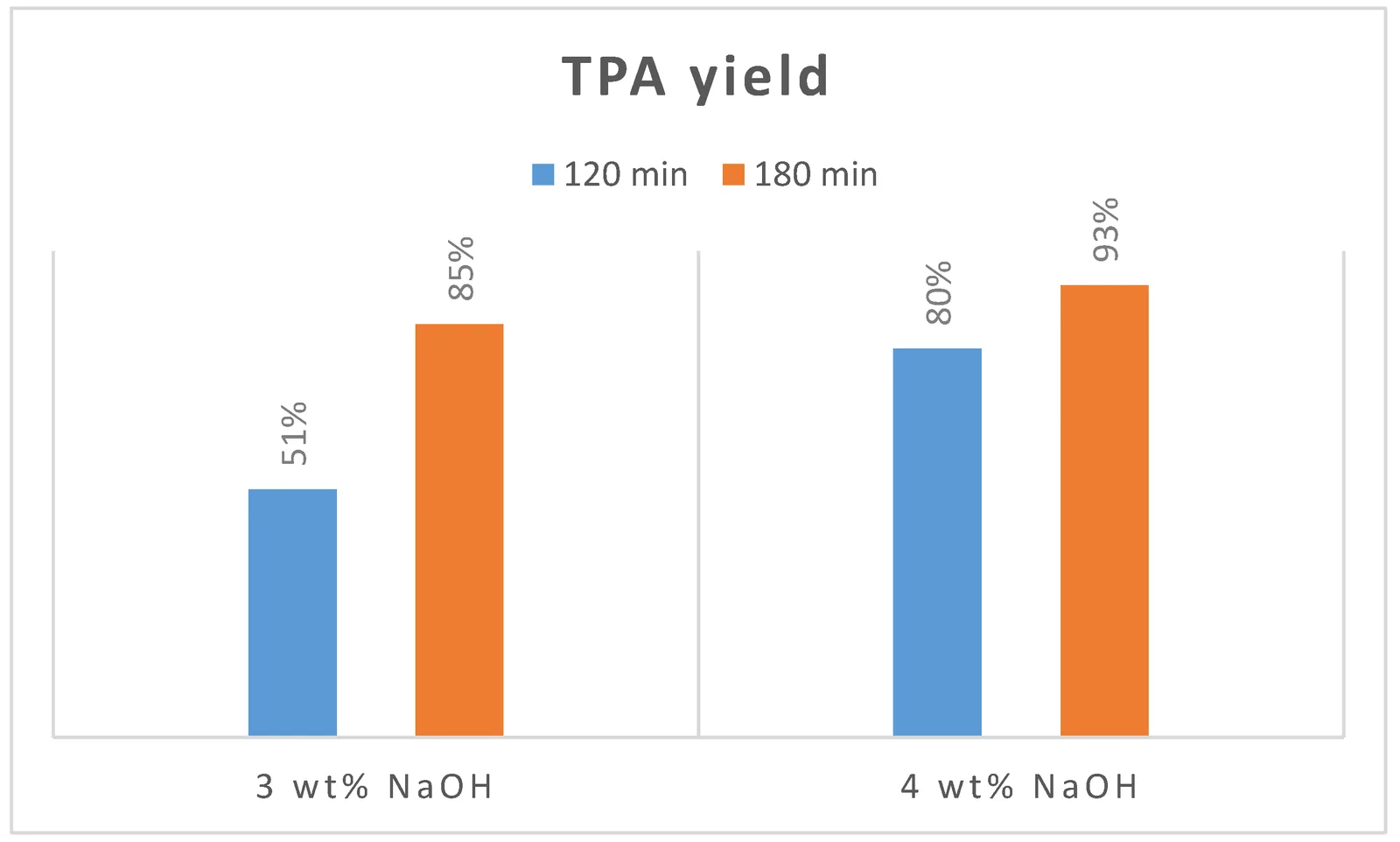
Effect of NaOH concentration on the TPA yield obtained from transparent PET under ohmic heating (ΩH) at reaction times of 120 and 180 min. Experiments were performed using PET flakes (0.2500 g) in 50 mL of EtOH/H_2_O (1:1, *v*/*v*) containing 3 or 4 wt% NaOH.

As shown in Figure 1, both reaction time and NaOH concentration influence TPA yield under ohmic heating conditions. Increasing the NaOH concentration from 3 to 4 wt% produced a significant improvement at 120 min, increasing the TPA yield from 51% to 80%, whereas a smaller increase was observed after 180 min (85% to 93%).

Overall, these results demonstrate that ohmic heating constitutes a viable alternative to conventional and microwave heating for PET hydrolysis, offering high TPA yields and allowing process optimization by adjusting the NaOH concentration, which is an electrolyte and the base of the reaction, and reusing the reaction medium. Furthermore, since ohmic heating is internal to the reaction mixture, as it depends on ion movement between the two electrodes, energy losses are minimized, making the method more energy-efficient.

TPA recovered from different PET sources was characterized using solid-state powder X-ray diffraction (PXRD) and also by liquid-state ^1^H NMR analysis.

**Figure 2:**
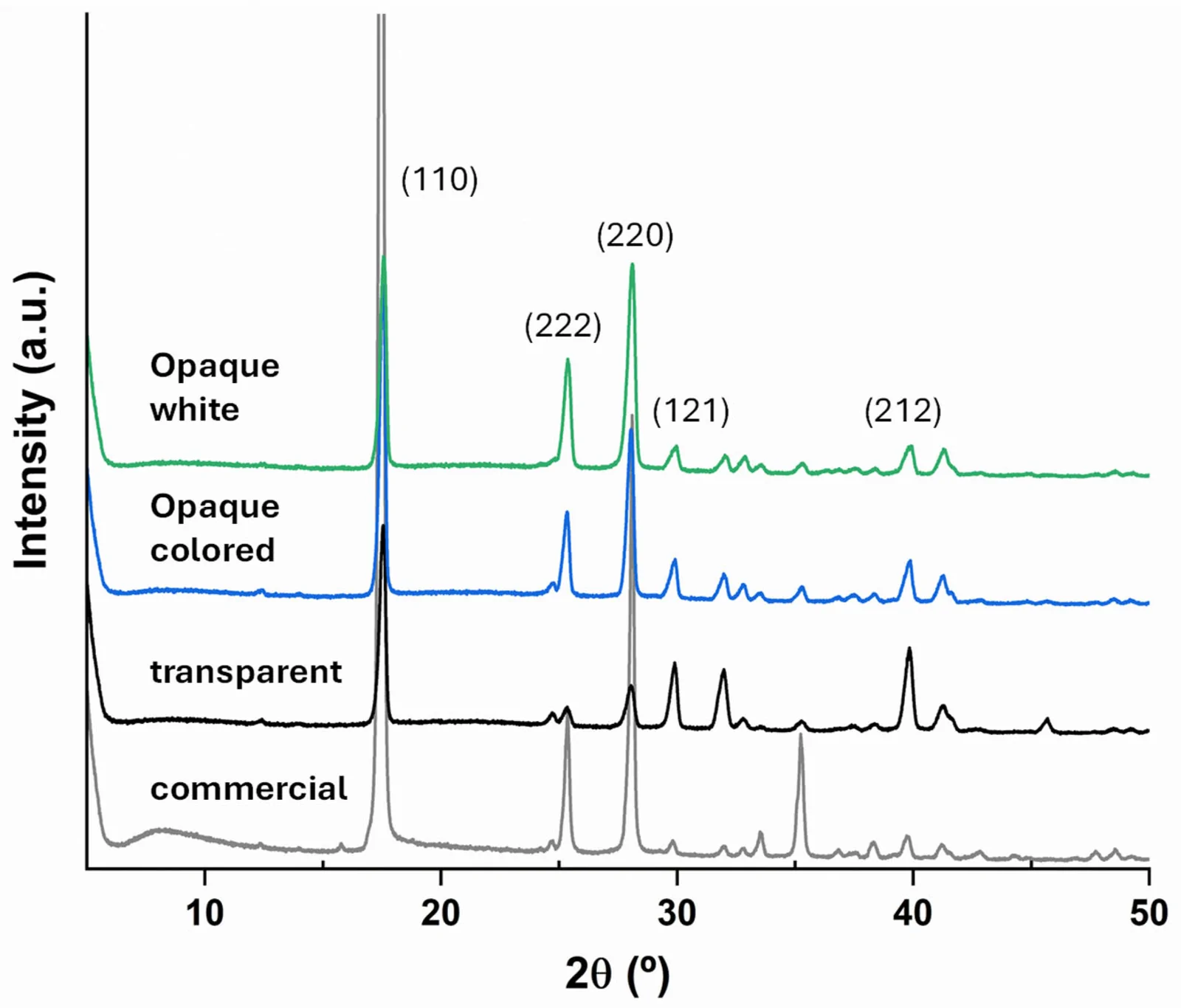
Powder X-ray diffraction patterns of terephthalic acid (TPA) from a commercial sample (commercial) and recovered from post-consumer PET after treatment using ohmic heating, using PET flakes from transparent bottles (transparent), colored bottles (opaque colored) and white bottles (opaque white).

Figure 2 shows the powder diffraction patterns of the TPA recovered from the different PET sources in comparison with the commercial one. The main diffraction reflections observed at approximately 17°, 25°, 28°, 32° and 40° can be assigned to the (110), (222), (220), (121) and (212) planes, respectively. These reflections are in good agreement with those reported for PET-derived TPA confirming the successful recovery of crystalline terephthalic acid [34,35,36].

Although the diffraction peak positions are essentially conserved among the samples, differences in relative peak intensities are observed, particularly for the reflections in the range 25–42°. Such differences may be associated with variations in crystallite morphology and/or preferred orientation during TPA precipitation and recovery, rather than indicating the formation of a different crystalline phase [34,36].

These findings are consistent with the ^1^H NMR spectra recorded in DMSO-d_6_ on a 400 MHz spectrometer. As shown in Figures S3, S5 and S6, TPA samples obtained from the alkaline hydrolysis of transparent, opaque white, and opaque colored PET flakes exhibited nearly identical spectral profiles. In all cases, the characteristic resonances of terephthalic acid were observed without evidence of significant impurities, confirming that the chemical structure of TPA was preserved during the hydrolysis and recovery processes (see Supplementary Materials).

### 2.2. Preparation of Binary Supramolecular Solids

TPA recovered from PET hydrolysis represents a valuable building block for the preparation of new materials. PET-derived TPA has already been explored as a linker for the synthesis of coordination materials, particularly metal-organic frameworks (MOFs) [35,37]. Materials prepared by supramolecular self-assembly offer advantages resultant from the reversibility of noncovalent interactions, which endows unique features such as stimuli-responsiveness, morphological variability or self-healability [29]. However, to the best of our knowledge, the use of TPA recovered from PET waste for the preparation of binary organic supramolecular solids has not yet been reported. Therefore, as a proof of concept for the further valorization of PET-derived TPA and to demonstrate its suitability for this type of application, two binary supramolecular solids were prepared by combining the recovered TPA with the pyridine derivatives 4-(dimethylamino)pyridine (DMAP) and 4-aminopyridine (AP). These compounds were selected because both exhibit high basicity due to the electron-donating effect of the amino group at the para position, which increases the electron density at the pyridine ring. DMAP is, however, more basic than AP owing to the additional electron-releasing inductive effect of its two methyl groups, which further stabilizes the corresponding protonated species. Consequently, both pyridine derivatives are expected to interact strongly with the carboxylic acid groups of TPA.

Inspired by the structures described by Mohamed et al. [27], the binary supramolecular assembly of PET-derived TPA with DMAP and AP was obtained through crystallization in methanol, resulting in compounds **1** and **2**, respectively. Transparent angular crystals were obtained for compound **1** after one week, while compound 2 showed transparent crystals with irregular rounded morphology in three days. The differences in crystalline form indicate that the nature and basicity of the pyridine co-former influence the crystallization behavior and supramolecular organization of the resulting solids. These materials were subsequently subjected to structural and morphological characterization.

**Figure 3:**
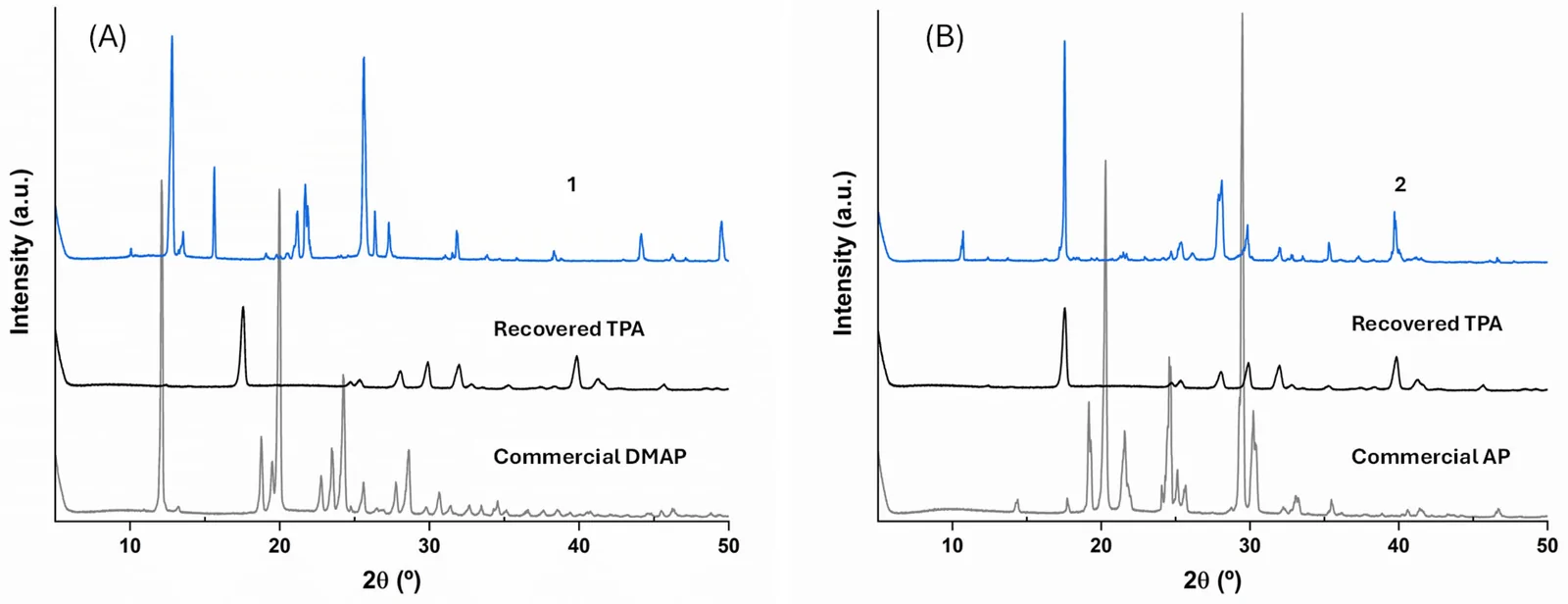
Powder X-ray diffraction patterns of (**A**) commercial 4-dimethylaminopyridine (Commercial DMAP), recovered terephthalic acid from transparent PET (Recovered TPA) and binary supramolecular solid **1**; (**B**) commercial 4-aminopyridine (Commercial AP); recovered terephthalic acid from transparent PET (Recovered TPA) and binary supramolecular solid **2**.

Figure 3 shows the PXRD patterns of the binary solids **1** and **2** in comparison with their respective reactants. The PXRD pattern of compound **1** (Figure 3A) differs markedly from those of its starting materials, TPA and DMAP, particularly in the relative intensities and distribution of the characteristic reflections. The absence of a simple superposition of the diffraction patterns of the two components is consistent with the formation of a new crystalline binary phase. This is further supported by the supramolecular structure obtained in this work, based on the assembly of two protonated DMAP units and a fully deprotonated TPA unit, which is in accordance with previous crystallographic studies [27].

The PXRD pattern of solid **2** (Figure 3B) differs from those of recovered TPA and commercial AP, despite retaining several reflections in the characteristic diffraction regions of TPA. Notably, significant changes in the relative intensities of these reflections are observed, together with additional diffraction features, indicating a different crystalline packing arrangement. These results are consistent with the formation of TPA–AP interactions leading to the formation of an ionic/hydrogen-bonded supramolecular solid, as reported for related 4-aminopyridine/terephthalate systems [38].

**Figure 4:**
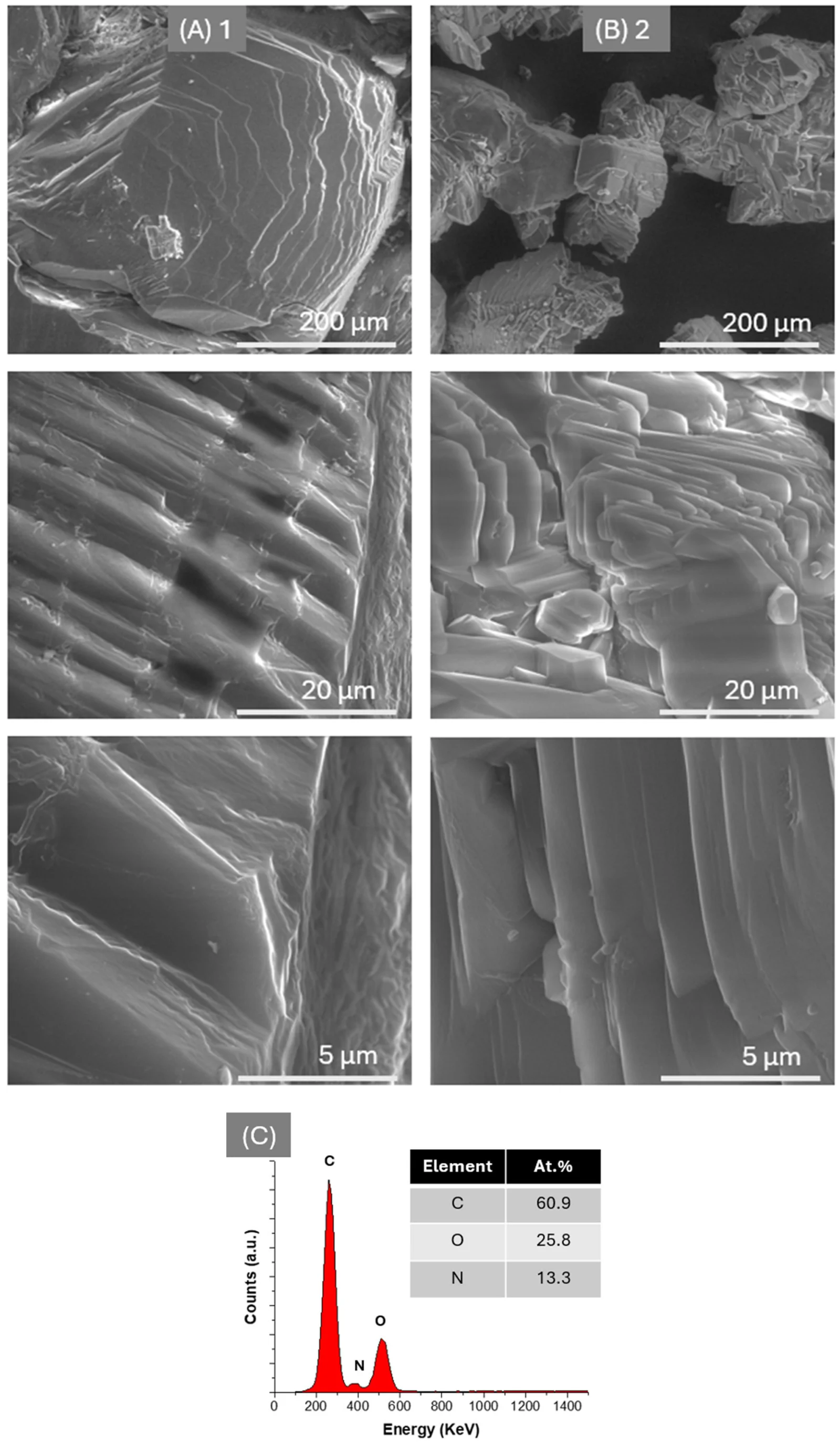
Morphological characterization and chemical composition of binary solids: SEM images at different magnifications of (**A**) Compound **1** and (**B**) Compound **2**; (**C**) EDS spectrum of **1** and microanalysis (at. %) in the inset.

SEM images of materials **1** and **2** are shown in Figure 4A and Figure 4B, respectively. Both materials display a comparable inherent morphological organization, albeit with larger particle sizes for **1** relative to **2**. Observation across different magnifications confirms a hierarchical architecture where smaller sub-units construct larger structural aggregates. Both materials exhibit stacked, overlapping sheet-like motifs reminiscent of ‘books on a bookshelf’, where micrometer-scale particles are formed by sheets of nanometric thickness. Supplementary SEM micrographs of **1** are presented in Figure S7.

Hierarchical structuring is a characteristic feature of self-assembled binary materials [31], driven by multiple supramolecular forces acting over different distance ranges (van der Waals, π–π staking, hydrogen bonding or electrostatic/ionic) [39]. Confirmation of the binary arrangement is provided by the single-crystal X-ray diffraction analysis presented below.

EDS and semi-quantitative elemental analysis of **1** reveal atomic percentages of 61% C, 13% N and 26% O (Figure 4C). This composition corroborates the inclusion of TPA and DMAP units, whereas solvent (methanol) entrapment within the supramolecular structure accounts for the increased O/N ratio.

**Figure 5:**
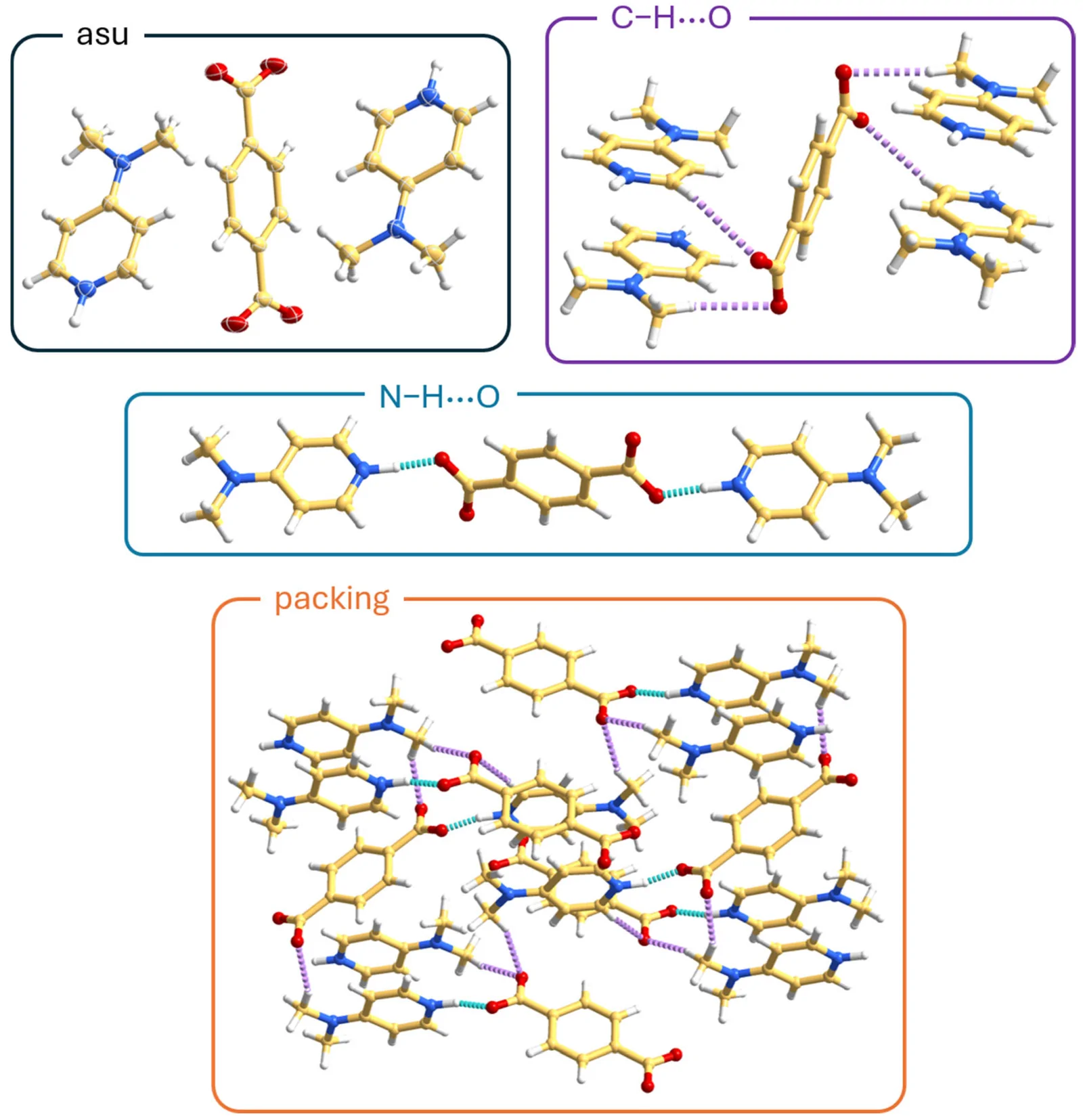
Details of the crystalline structure of the binary supramolecular solid (compound **1**) obtained from self-assembly of terephthalic acid obtained from PET bottles and 4-(dimethylamino)pyridine. Elements color: O—red; C—yellow; N—blue; H—white; C–H···O and N–H···O represented as violet and green-blue dotted lines.

The controlled self-assembling reaction between the terephthalic acid obtained from PET bottles and 4-(dimethylamino)pyridine in methanol yielded suitable single crystals for X-ray diffraction analysis. The solid-state crystalline structure was determined in the monoclinic space group P21/n, unequivocally confirming the preparation of a binary supramolecular structure (Figure 5). This supramolecular structure is based on the assembly of two protonated 4-(dimethylamino)pyridine units (two cationic units) and a terephthalate unit (fully deprotonated). The asymmetric unit (asu) reveals a terephthalate unit and two protonated 4-(dimethylamino)pyridine molecules arranged in opposite directions. In fact, each terephthalate unit closely interacts by strong N–H···O hydrogen bonds with two adjacent 4-(dimethylamino)pyridine molecules. Furthermore, several C–H···O weak hydrogen bonds lead to a three-dimensional supramolecular network, reinforcing the structural stability of this binary supramolecular solid (Figure 5; see Table 2 for geometric details about the hydrogen bonding interactions).

## 3. Materials and Methods

All reactants and solvents used in this work were purchased from commercial suppliers and were used as received.

Three methodologies were employed to accomplish the alkaline hydrolysis of PET: (a) conventional heating of the reflux (83 °C) using oil bath on an IKA C-MAG HS 7 stirring hotplate; (b) microwave-assisted hydrolysis was carried out in a CEM Discovery Labmate circular single-mode cavity instrument (300 W max magnetron power output) from the CEM Corporation, using reflux (83 °C) under open-vessel conditions and (c) Ohmic heating [23] comprising the following elements: the AC power supply; the signal generator; the heating reactor cell (50 mL glass vessel) containing one pair of stainless steel (316) rectangular electrodes (40 mm length × 25 mm wide; 1 mm thickness) located at a fixed distance (23 mm); temperature sensor (K type thermocouple); the data logger system; PC computer; data acquisition and control software package. The AC voltage (VAC) ranges from 5 to 100 VAC, and the typical working frequency is 25 kHz. The typical power applied to the reactor in the reflux regime is 250 W.

Nuclear magnetic resonance (NMR) spectra were recorded on a Bruker Avance III 400 spectrometer (400.15 MHz for ^1^H and 100.63 MHz for ^13^C). Chemical shifts (δ) are reported in ppm and coupling constants (*J*) in Hz, relative to tetramethylsilane (TMS) as the internal standard.

The PXRD patterns were acquired using a Bruker D6 PHASER diffractometer equipped with a Cu Kα radiation source (λ = 1.5406 Å), over a 2θ range of 5–50°, with a step size of 0.01° and a counting time of 0.2 s per step.

SEM images and EDS spectra were obtained using a high-resolution (Schottky) Environmental Scanning Electron Microscope and Electron Backscattered Diffraction Analysis (Quanta 400 FEG ESEM/EDAX Genesis X4M) typically operated at 15 kV, with around a 10 mm working distance. All SEM data shown are secondary electron images obtained at low vacuum and with no sample coating.

Single crystals of compound **1** were manually harvested from the crystallization vial and immersed in highly viscous inert vacuum oil, with degradation instigated by the evaporation of the solvent [27]. A suitable crystal was mounted on a Hampton Research CryoLoops with the help of a Stemi 2000 stereomicroscope equipped with Carl Zeiss lenses. The X-ray diffraction data were collected on a Rigaku Oxford Diffraction XtaLAB Synergy-i diffractometer using a monochromatized microfocus PhotonJet-I MoKα radiation source (λ = 0.71073 Å) and a HiPix3000 Bantam detector, also equipped with a Cryostream 800 (Oxford Cryosystems). Evaluation, integration, and reduction of the diffraction data were carried out with the CrysAlis Pro software suite [27]. The structure was solved using the algorithms implemented in SHELXT-2014 [27,40,41], allowing the direct location of almost all the heaviest atoms. The remaining non-H atoms were located from difference Fourier maps calculated from successive full-matrix least squares refinement cycles on *F*^2^ using SHELXL-v.2019 [27,40,42] and all of them were successfully refined using anisotropic displacement parameters.

H-atoms bound to carbon were located at model geometrical positions using suitable HFIX instructions in SHELXL: *43* for the aromatic and *137* for the CH_3_ groups belonging to the two distinct organic molecules. All these atoms were included in subsequent refinement cycles in riding-motion approximation with isotropic thermal displacement parameters (*U*_iso_) fixed at 1.2 or 1.5 × *U*_eq_ of the attached carbon atom. The protonated H atom of the 4-(dimethylamino)pyridine was markedly visible in the difference Fourier maps and included in subsequent refinement stages with the N−H distance restrained to ensure a chemically reasonable geometry for this group.

Table 3 summarizes relevant information about the crystal data, single-crystal X-ray data collection and structure refinement details. A CIF has been deposited with the Cambridge Crystallographic Data Centre as supplementary publication data N°. CCDC-2587431. These data can be obtained online at https://www.ccdc.cam.ac.uk/structures/.

**Preparation of PET flakes:** Three types of PET flakes were obtained from post-consumer PET bottles: (a) transparent; (b) opaque white and (c) opaque colored. These bottles were washed, dried, cut with scissors, and finally ground in a food processor (Figures S1 and S2; see Supplementary Materials for details). The particle size distribution of the PET flakes was determined using a Retsch AS 200 sieve shaker. Sieving was performed for 10 min at an amplitude setting of 80, using sieves with the following mesh sizes: 2 mm, 1 mm, 0.5 mm, 0.250 mm, 0.125 mm and 0.063 mm (Table S1).

**Alkaline hydrolysis of transparent PET flakes (oil bath method):** In a 50 mL round-bottom flask fitted with a reflux condenser, NaOH powder (2.840 g) was dissolved in 50 mL of an EtOH/H_2_O mixture (1:1, *v*/*v*), and transparent PET flakes (0.2500 g) were added. The mixture was refluxed at 85 °C using an oil bath under magnetic stirring for 90 min. After heating, the reaction mixture was filtered to remove unreacted PET. Then, HCl 37% was added dropwise to the filtrate until it became cloudy and reached a pH below 1. The mixture was then allowed to cool to promote complete precipitation of terephthalic acid as a white precipitate. The terephthalic acid was filtered under vacuum, washed twice with ethanol, and dried in an oven at 60 °C for at least 24 h. The resulting terephthalic acid was weighed to determine the final yield of the reaction TPA (0.1617 g, 74% yield; Recovered PET (0.0150 g, 94% conversion).

**Alkaline hydrolysis of transparent PET flakes (microwave method):** In a 50 mL round-bottom flask fitted with a reflux condenser, NaOH powder (2.840 g) was dissolved in 50 mL of an EtOH: H_2_O mixture (1:1, *v*/*v*), and transparent PET flakes (0.2500 g) were added. The mixture was placed in the microwave cavity, and it was refluxed at 85 °C for 90 min using 300 W maximum power. After heating, the reaction mixture was filtered to remove unreacted PET. Then, 37% HCl was added dropwise to the filtrate until it became cloudy and reached a pH below 1. The mixture was then allowed to cool to promote complete precipitation of terephthalic acid as a white precipitate. The terephthalic acid was filtered under vacuum, washed twice with ethanol, and dried in an oven at 60 °C for at least 24 h. The resulting terephthalic acid was weighed to determine the final yield of the reaction TPA (0.0896 g, 41% yield; Recovered PET (0.1275 g, 49% conversion).

**Alkaline hydrolysis of three type of PET flakes (ohmic method):** PET flakes, including transparent, opaque white or opaque colored flakes (0.2500 g) were added to a solution of NaOH (1.900 g) dissolved in 50 mL of an EtOH/H_2_O mixture (1:1, *v*/*v*). The reaction mixture was heated under ohmic heating (25 kHz frequency, 400 mV amplitude) under reflux for 180 min. During the reaction, the following operating parameters were recorded: temperature, 82.6 °C; AC voltage, 5.47 V; AC current, 4.94 A; impedance, 1.1 Ω; and load power, 27 W. After heating, the reaction mixture was filtered to separate any unreacted PET. For opaque colored PET samples, activated charcoal (approximately one spatula tip) was added to the filtrate, which was then stirred at room temperature for 10 min and filtered again to remove the residual purple pigment. Concentrated HCl (37%) was subsequently added dropwise to the filtrate until the solution became turbid and the pH decreased below 1. The mixture was then allowed to cool to promote complete precipitation of terephthalic acid as a white precipitate. The resulting white precipitate was collected by filtration, washed twice with ethanol, and dried in an oven at 60 °C for at least 24 h. The recovered TPA was weighed and used to calculate the product yield. Transparent PET: TPA (0.2032 g, 93% yield; Recovered PET (complete conversion). Opaque White PET: TPA (0.1644 g, 74% yield); Recovered PET (0.0335 g, 87% conversion). Opaque Colored PET: TPA (0.1329 g, 58% yield); Recovered PET (0.0790 g, 70% conversion). The TPA obtained was subsequently characterized by ^1^H NMR (Figures S3, S5 and S6) and ^13^C NMR (Figure S4).

For the sequential transparent PET addition experiment: Transparent PET flakes (0.2500 g), NaOH (1.900 g), and 50 mL of an EtOH/H_2_O mixture (1:1, *v*/*v*) were heated under the same ohmic conditions for 120 min. Subsequently, another 0.2500 g of PET flakes were added, and the reaction continued for 180 min. The reaction processing was carried out as previously described, affording 0.3321 g of TPA. Considering the use of 0.5000 g of PET, the TPA yield obtained was 76%; Recovered PET (0.1200 g, 76% conversion).

**Compound 1**: TPA (0.1 mmol) and 4-(dimethylamino)pyridine (0.1 mmol, Sigma-Aldrich, 98%) were first ground together in a mortar to ensure homogeneous mixing. The resulting powder was dissolved in the minimum volume of methanol required for complete dissolution (35.0 mL), and the solution was left to crystallize slowly under refrigerated conditions. After one week, transparent angular crystals suitable for further characterization were obtained.

**Compound 2**: TPA (0.1 mmol) and 4-aminopyridine (0.1 mmol, Sigma-Aldrich, 98%) were first ground together in a mortar. The homogeneous mixture was dissolved in methanol (5.5 mL) and allowed to crystallize slowly under refrigerated conditions. After approximately three days, transparent crystals displaying an irregular rounded morphology were observed and subsequently isolated for characterization.

## 4. Conclusions

In this study, we explored the hydrolysis of PET by ohmic heating and compared it with conventional and microwave heating methods. From the results obtained, we can conclude that the ohmic heating treatment stands out for its high efficiency in PET hydrolysis, offering excellent TPA yields and allowing process optimization through adjustment of the NaOH concentration and reuse of the reaction medium.

Two binary supramolecular structures (compounds **1** and **2**) were successfully synthesized by combining recovered TPA with pyridine derivatives: 4-(dimethylamino)pyridine (DMPA) and 4-aminopyridine (AP), in methanol at low temperature. Particularly for compound **1**, single-crystal X-ray diffraction analysis revealed a three-dimensional supramolecular network stabilized by robust hydrogen-bonding interactions between TPA and DMAP units, corroborated by the overlapping lamellae’s hierarchical supramolecular structure observed by SEM.

Overall, the successful construction of these supramolecular architectures demonstrates that recycled TPA can serve as a valuable platform for advanced functional materials, providing a sustainable strategy for high-value PET upcycling.

## Figures and Tables

**Table 1:** PET conversion (%) and TPA yield (%) under different heating methodologies and reaction conditions ^a^.

Entry	Type of PET	Heating Method	NaOH (wt%)	Time (min)	PET Conversion (%)	TPA Yield (%)
1	Transparent	Oil bath	6	90	94	74
2	Transparent	MW	6	90	49	41
3 ^b^	Transparent	ΩH	6	n.d.	n.d.	n.d.
4	Transparent	ΩH	3	120	81	51
5	Transparent	ΩH	3	180	93	85
6	Transparent	ΩH	4	120	89	80
7	Transparent	ΩH	4	180	complete	93
8 ^c^	Transparent	ΩH	4	120 + 180	76	76
9	Opaque white	ΩH	4	180	87	74
10	Opaque colored	ΩH	4	180	70	58

^a^ General experimental conditions: PET flakes (0.2500 g) in 50 mL of EtOH/H_2_O (1:1, *v*/*v*) containing NaOH (3–6 wt%) were heated using oil-bath, microwave (MW) or ohmic heating (ΩH, 25 kHz) for the indicated reaction time; ^b^ the measured conductivity was 72.3 mS cm^−1^, which caused a drop in voltage. This exceeds the average conductivity value of 50 mS cm^−1^, which is typically operated in our ohmic reactor; ^c^ sequential PET addition experiment: PET flakes (0.2500 g) were heated for 120 min, followed by addition of a second 0.2500 g PET portion and heating for a further 180 min under identical conditions.

**Table 2:** Geometric information (distances in Å and angles in degrees) for the D–H···A hydrogen bond interactions for compound **1**.

D–H···A	*d* (H···A)	*d* (C···A)	∠(DHA)
C(5)−H(5)∙∙∙O(1) ^i^	0.93	2.46	3.386(3)
C(10)−H(10C)∙∙∙O(1) ^ii^	0.96	2.58	3.473(4)
N(1)−H(7)∙∙∙O(2) ^iii^	1.05(3)	1.54(3)	2.577(3)

Symmetry transformations used to generate equivalent atoms: ^i^ *x* + 1, *y*, *z*; ^ii^ −*x* + 1, −*y*, −*z* + 1; ^iii^ *x* + 1/2, −*y* + 1/2, *z* − 1/2.

**Table 3:** Crystal and structure refinement data for compound **1**.

Chemical formula	C_22_H_26_N_4_O_4_
M*_r_*	410.47
Crystal description	Colourless plate
Crystal size/mm	0.22 × 0.09 × 0.02
Crystal system, space group	Monoclinic, *P*2_1_/*n*
*a*/Å	7.0164(12)
*b*/Å	15.777(3)
*c*/Å	9.7721(14)
*β*/°	110.93(2)
Volume/Å^3^	1010.4(3)
*Z*	2
ρ*_calculated_*/g cm^−3^	1.349
*F*(000)	436
*μ*/mm^−1^	0.095
*θ* range/°	4.042 to 26.367
Index ranges	−8 ≤ *h* ≤ 8 −19 ≤ *k* ≤ 19 −12 ≤ *l* ≤ 7
Reflections collected	7361
Independent reflections	1738 (*R*_int_ = 0.0324)
Final *R* indices [*I* > 2σ(*I*)]	*R*_1_ = 0.0511; w*R*_2_ = 0.1340
Final *R* indices (all data)	*R*_1_ = 0.0823; w*R*_2_ = 0.1470
Δ*ρ_max_* and Δ*ρ_min_*/e.Å^−3^	0.193 and −0.178

## Data Availability

The original contributions presented in this study are included in the article/Supplementary Material. Further inquiries can be directed to the corresponding author.

## Supplementary Materials

The following supporting information can be downloaded at: https://www.mdpi.com/article/10.3390/molecules31203698/s1, Table S1: The particle size distribution of the PET flakes determined using a Retsch AS 200 sieve shaker. Figure S1: Photographs of PET bottles after washing and drying (A); after being cut with scissors (B) and after being crushed in a food processor (C). Figure S2: Flowchart for the recovery of terephthalic acid from PET bottles. Figure S3: ^1^H NMR (400 MHz, DMSO-d6) of TPA obtained from alkaline hydrolysis of transparent PET flakes. Figure S4: ^13^C NMR (100 MHz, DMSO-d6) of TPA obtained from alkaline hydrolysis of transparent PET flakes. Figure S5: ^1^H NMR (400 MHz, DMSO-d6) of TPA obtained from alkaline hydrolysis of opaque white PET flakes. Figure S6: ^1^H NMR (400 MHz, DMSO-d6) of TPA obtained from alkaline hydrolysis of opaque colored PET flakes. Figure S7: Complementary SEM images of material 1 at different magnifications (A–C).
