# Supplementary material for: Ohmic Heating as a Strategy for Upcycling PET Plastic Waste for New Supramolecular Structures
Source: Molecules. 2026 Oct 15;31(20):3698. doi: 10.3390/molecules31203698 (PMC13707624; doi:10.3390/molecules31203698)
Supplement: Supplementary file 1 [file molecules-31-03698-s001.zip › molecules-4596344-supplementary.pdf]

Supporting information for

Ohmic Heating as a Strategy for Upcycling PET Plastic Waste for New Supramolecular Structures

Susana Guimarães, Susana L. H. Rebelo, Sandra Gago, Maria A. Salgado, Luís M. N. B. F. Santos, Salete S. Balula, Luís Cunha-Silva and Ana M. G. Silva

Contents

Preparation and characterization of PET flakes 2
NMR analysis 4
SEM complementary results 6

Preparation and characterization of PET flakes

Each PET bottle was visually inspected. If labels, glue residue, or other contaminants were observed, these materials were completely removed. Labels, caps, etc., were removed manually. In all cases, bottles were washed with hot water and dish soap, rinsed, and dried. Subsequently, the clean PET bottles were cut with scissors and ground in a kitchen grinder. The particle size distribution of the PET flakes was determined using a Retsch AS 200 sieve shaker. Sieving was performed for 10 min at an amplitude setting of 80, using sieves with the following mesh sizes: >2 mm, 1–2 mm, 0.5–1 mm, 0.250–0.500 mm, 0.125–0.250 mm and 0.063–0.125 mm and <0.063 mm (Table S1).

Table S1. *The particle size distribution of the PET flakes determined using a Retsch AS 200 sieve shaker.*

SIEVE PLATE (MM)	TRANSPARENT PET %	OPAQUE WHITE PET %	OPAQUE COLORED PET %
**>2**	92.90	76.20	81.54
**1–2**	5.10	16.27	11.18
**0.5–1**	1.40	5.17	4.73
**0.250–0.500**	0.40	1.47	1.91
**0.125-0.250**	0.20	0.88	0.36
**0.063–0.125**	0.00	0.00	0.00
**<0.063**	0.00	0.00	0.00
**TOTAL**	100.0	99.99	99.72

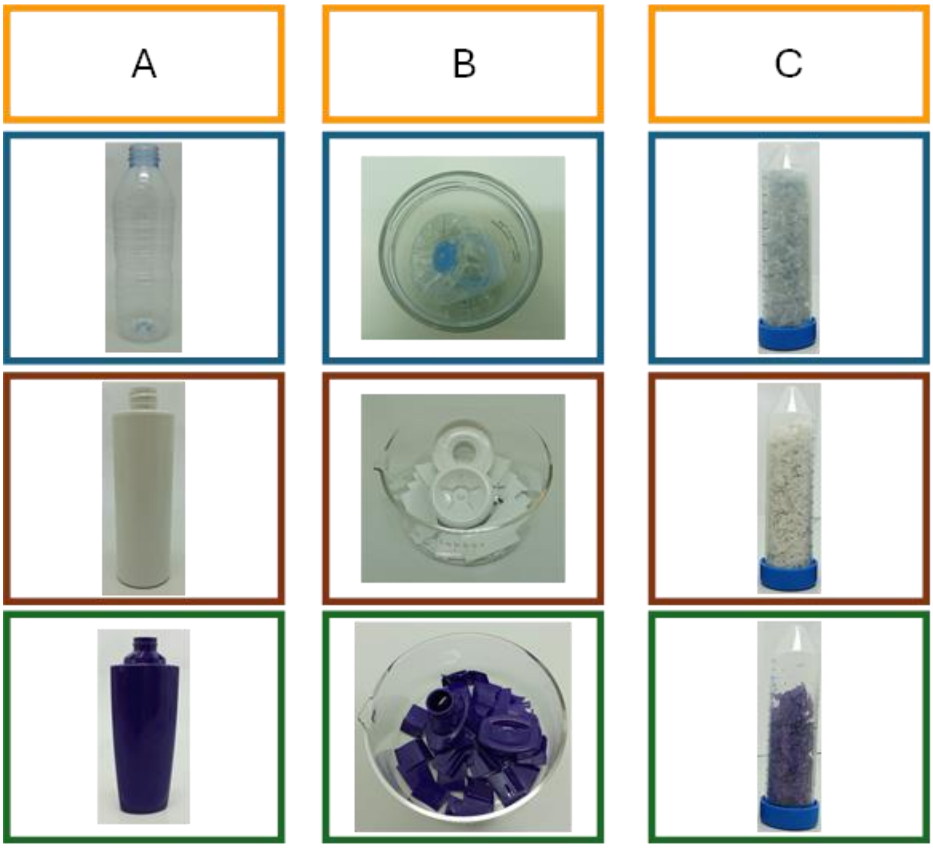

Figure S1. *Photographs of PET bottles after washing and drying (A); after being cut with scissors (B) and after being crushed in a food processor (C).*

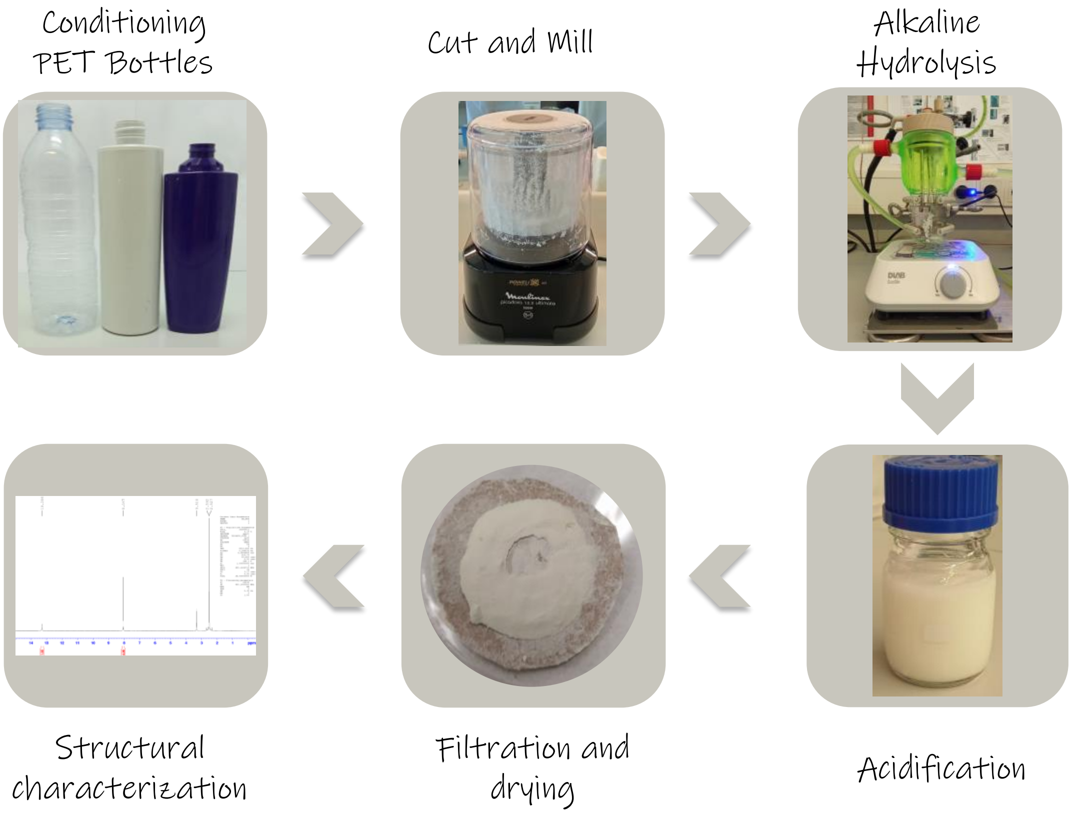

Figure S2. *Flowchart for the recovery of terephthalic acid from PET bottles.*

NMR analysis

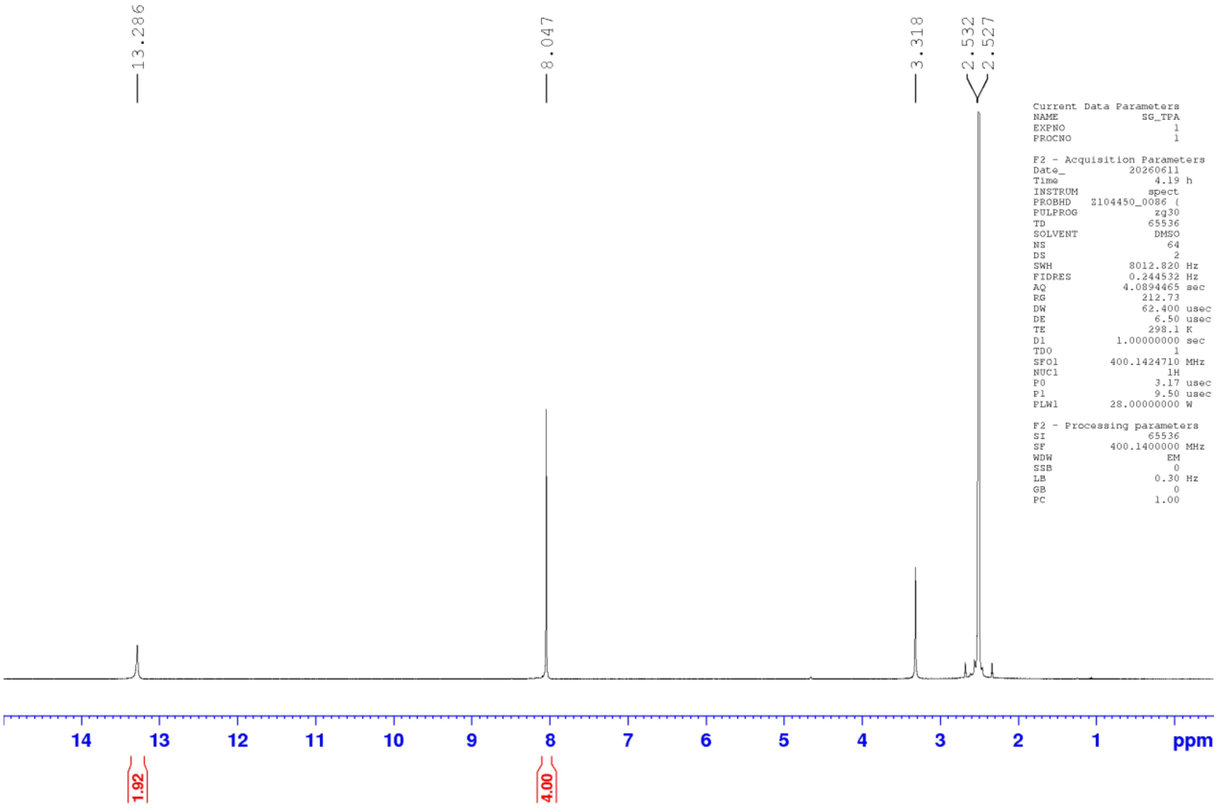

Figure S3. *^{1}H NMR (400 MHz, DMSO-d6) of TPA obtained from alkaline hydrolysis of transparent PET flakes.*

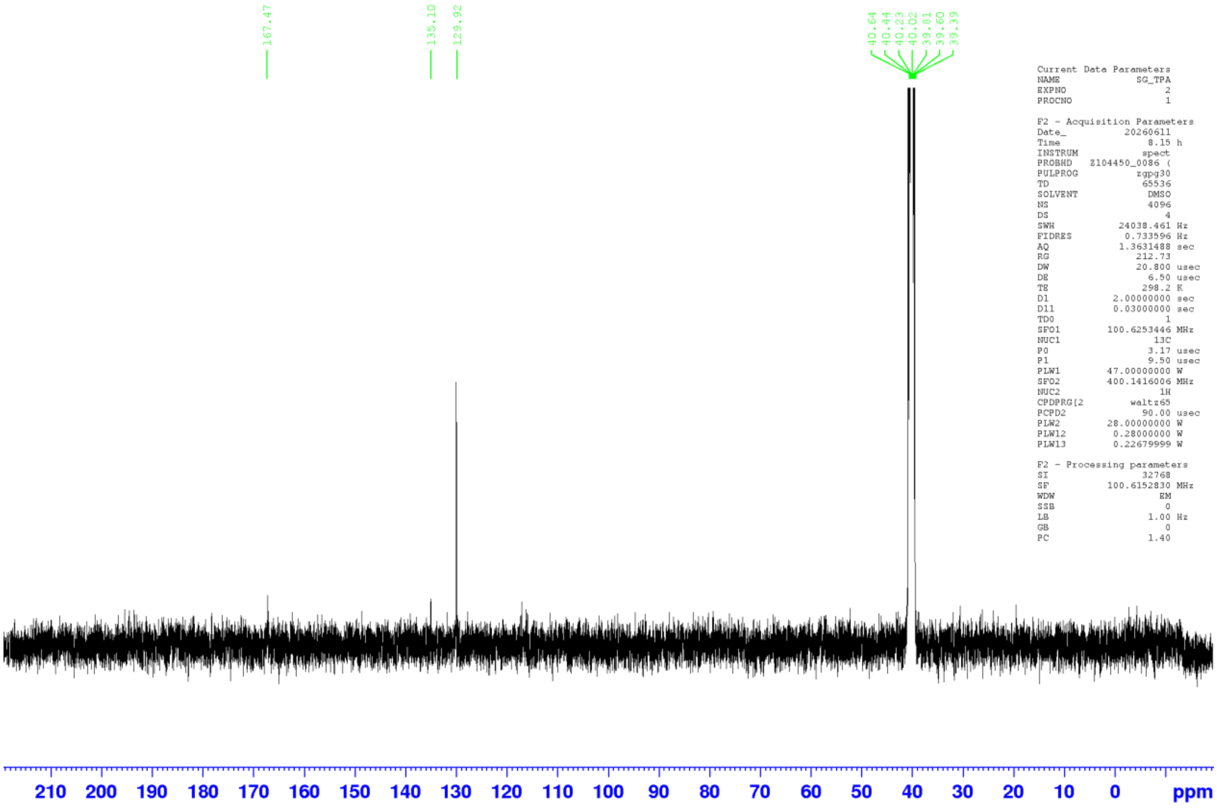

Figure S4. *^{13}C NMR (100 MHz, DMSO-d6) of TPA obtained from alkaline hydrolysis of transparent PET flakes.*

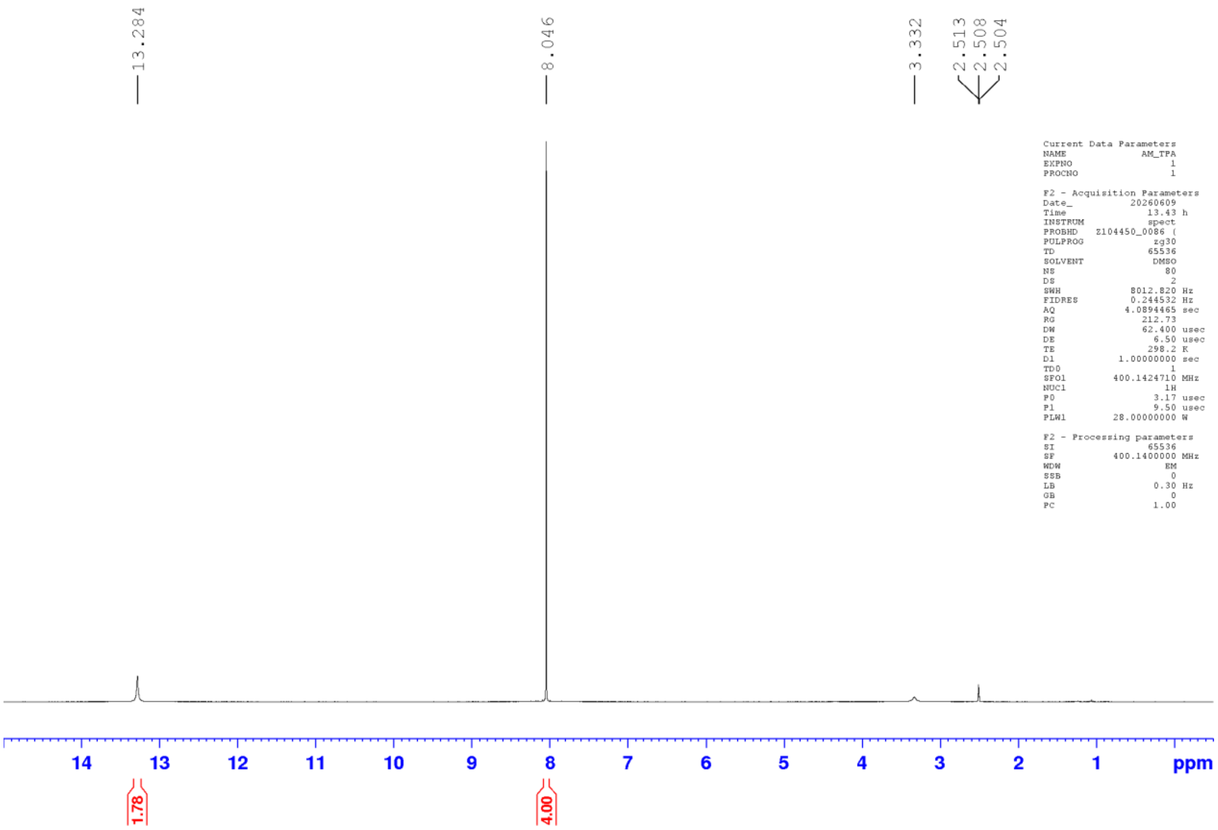

Figure S5. [1]*H NMR (400 MHz, DMSO-d6) of TPA obtained from alkaline hydrolysis of opaque white PET flakes.*

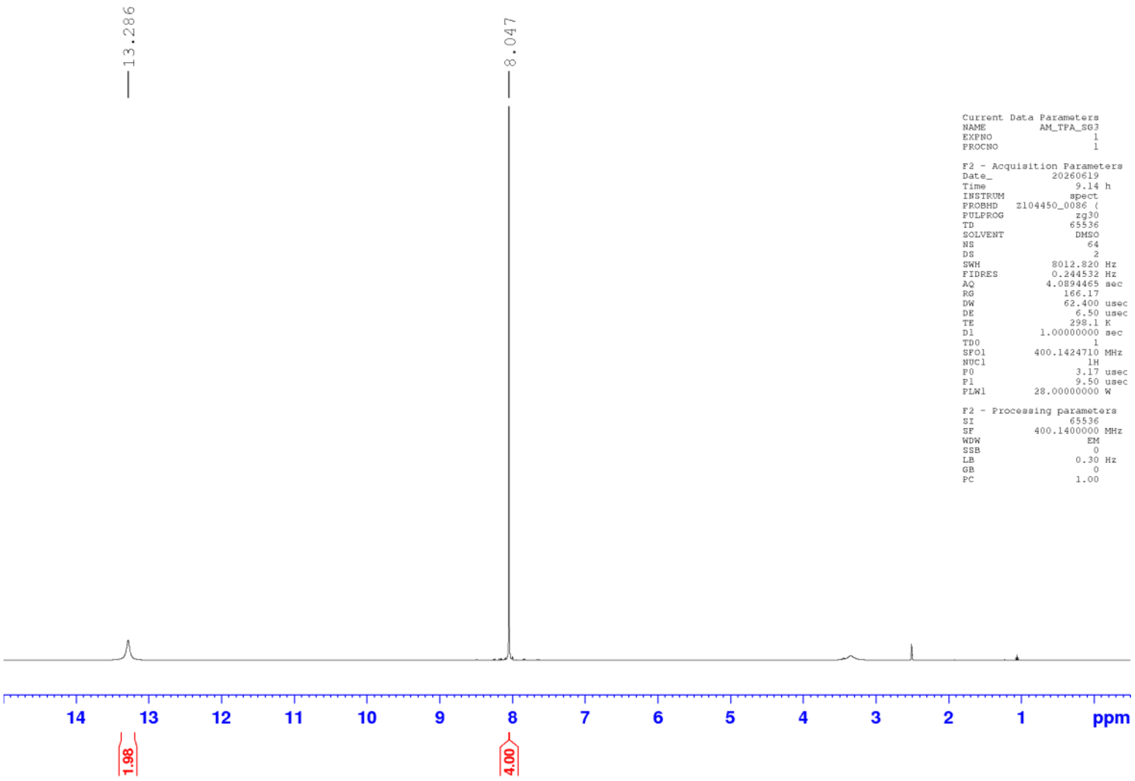

Figure S6. [1]*H NMR (400 MHz, DMSO-d6) of TPA obtained from alkaline hydrolysis of opaque colored PET flakes.*

SEM complementary results

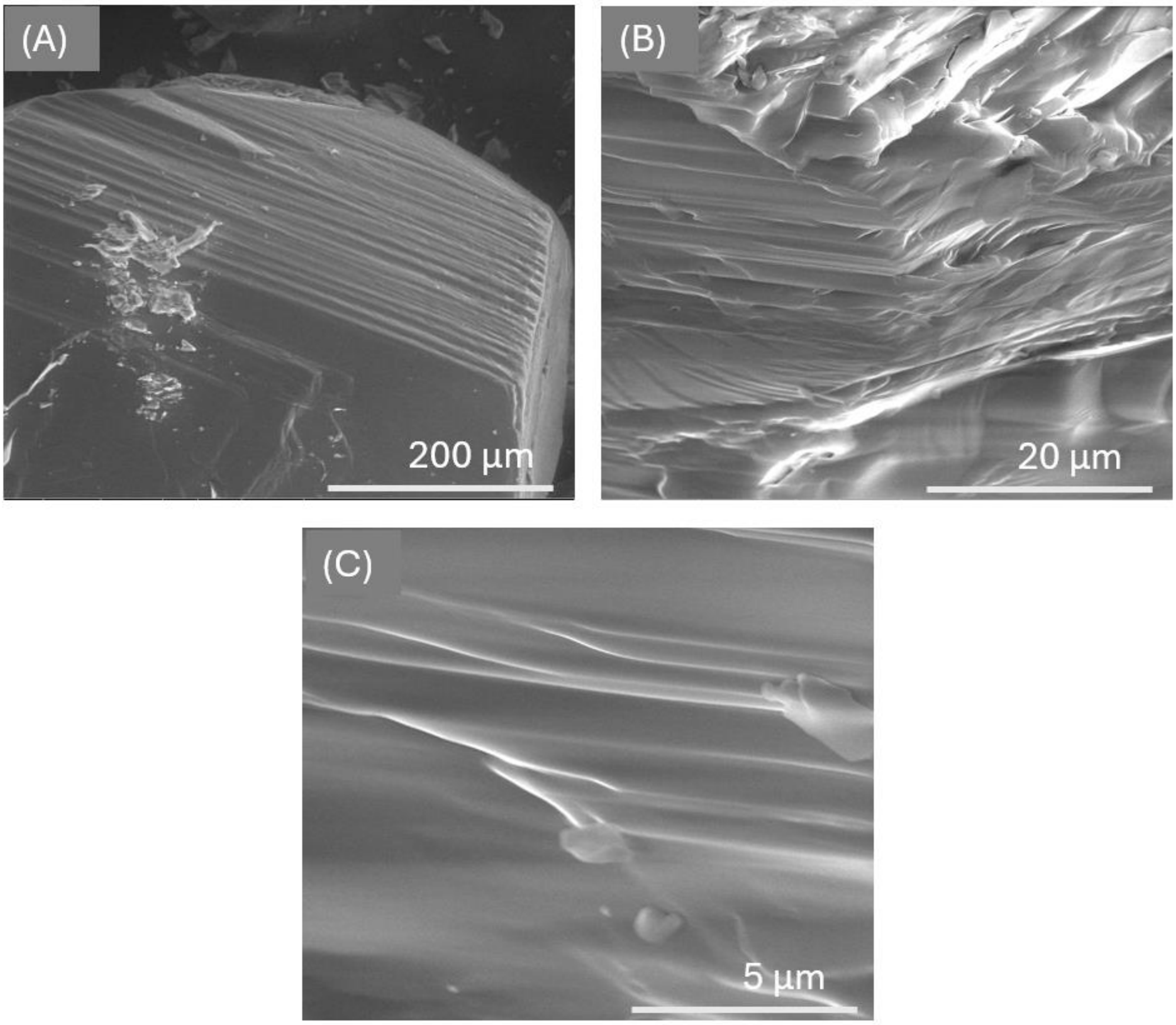

Figure S7. *Complementary SEM images of material* ***1*** *at different magnifications (A-C)*
